# Societal- and community-level strategies to improve social connectedness among older adults

**DOI:** 10.3389/fpubh.2023.1176895

**Published:** 2023-05-04

**Authors:** Matthew Lee Smith, Jillian Racoosin, Risa Wilkerson, Ronald Matthew Ivey, Louise Hawkley, Julianne Holt-Lunstad, Thomas K. M. Cudjoe

**Affiliations:** ^1^School of Public Health, Texas A&M University, College Station, TX, United States; ^2^Center for Community Health and Aging, Texas A&M University, College Station, TX, United States; ^3^Center for Health Equity and Evaluation Research, Texas A&M University, College Station, TX, United States; ^4^Foundation for Social Connection, Washington, DC, United States; ^5^Coalition to End Social Isolation and Loneliness, Washington, DC, United States; ^6^Global Initiative on Loneliness and Connection, Washington, DC, United States; ^7^Healthy Places by Design, Carrboro, NC, United States; ^8^The Human Flourishing Program, The Institute for Quantitative Social Sciences, Harvard University, Cambridge, MA, United States; ^9^Academic Research Centers, NORC at the University of Chicago, Chicago, IL, United States; ^10^Departments of Psychology and Neuroscience, Brigham Young University, Provo, UT, United States; ^11^Division of Geriatric Medicine and Gerontology, Johns Hopkins University School of Medicine, Baltimore, MD, United States

**Keywords:** social connectedness, social isolation, loneliness, community capacity, strategic initiatives, organizational collaboration

## 1. Introduction

Social disconnectedness is a complex and multi-faceted public health issue impacting individuals of all ages across the life-course. Social disconnectedness is characterized by the interrelated concepts of social isolation and loneliness stemming from limited contact or meaningful relationships with others, or related perceptions thereof. Older adults may be particularly at risk for social disconnectedness because they are more likely to live alone, experience loss or changes in their social networks (e.g., spouse, family, friends), and have chronic conditions and impairments (e.g., mobility, sensory, cognitive). In the United States, about 25% of older adults are considered to be socially isolated (1), which is an objective measure indicating the absence of a social network or the lack of social contact (2). Further, anywhere between 20% to 40% of older adults report moderate to severe loneliness (3–5), which can be described as the subjective, negative feeling from inadequate meaningful connections (6) or a lack of connection to other people despite the desire for more, or more satisfying, social relationships (7). People who feel they do not belong to majority social groups because of their gender identity, race, ethnicity, religion, language, or sexual orientation are at increased risk for social isolation, as are people living in rural areas, people with disabilities, immigrants, and individuals and families with financial struggles (8–11). The ramifications of social disconnectedness are vast and span poor physical (e.g., cardiovascular disease, stroke) (12–14) and mental (e.g., depression, anxiety) health outcomes, cognitive decline, risky health behaviors (e.g., substance use, physical inactivity, suicide), and all-cause mortality (2, 15–17).

Social connectedness is recognized as a core dimension of individual flourishing, health, wellbeing, and survival (18, 19). The longest longitudinal study of adults, the 75-year Harvard Study of Adult Development, found that an individual's satisfaction with their relationships was the greatest predictor of happiness and health (20, 21). Social connectedness has also been shown to be a key indicator of healthy aging later in life. Socially connected older adults are the core of an optimally functioning society (22). Living in socially connected communities can help older adults to thrive because it can increase neighborhood safety, strengthen resilience during societal crisis, encourage volunteerism, improve access to services and supports, and facilitate trust (23). Cognitive science demonstrates that friendships are critical for shared social pursuits of truth and that chronic forms of social isolation and loneliness contribute to distrust in social and political institutions (24).

While the consequences of social disconnectedness can be detrimental to the health, they may be symptoms of a fragmented and siloed society that obstructs and complicates efforts to build social connectedness for older adults (25). In this context, the purposes of this article are to: (a) describe societal-level challenges that foster social disconnectedness; and (b) provide opportunities and solutions to strengthen community capacity to foster social connectedness among older adults. This article brings together experts from public health, medicine, psychology, public policy, social sciences, and healthy community design to provide diverse perspectives through a unified lens to guide research, practice, and policy to drive community-level action.

## 2. Societal disconnectedness

Social connectedness is the degree to which an individual or population falls along the continuum of social connection, which includes (a) connections to others via the existence of relationships and their roles; (b) a sense of connection that results from actual or perceived support or inclusion; and c) the sense of connection to others that is based on positive relationship qualities (26, 27). Social connectedness is comprised of various interpersonal bonds (e.g., marriages, families, friendships)- bonds with strong (spouses, family, friends) and weak ties (infrequent, arms-length relationships), and various forms of participation in community life including memberships in civic, religious, social, and/or political organizations and networks that share common missions, interests, values, and beliefs (28). However, at times community systems and infrastructures can limit opportunities for interaction and participation, which can be detrimental to social connectedness.

In the context of public health, communities are “a group of people with diverse characteristics who are linked by social ties, share common perspectives, and engage in joint action in geographical locations or settings” (29). Communities are comprised of interrelated systems that provide services and programs to improve and maintain older adults' health and wellness. To support mental and physical health, these networks can facilitate the initiation, maintenance, and strength of interpersonal bonds and participation in community life. Spanning the aging services network, public health system, and healthcare sector, each organization serving older adults has a unique mission, set of offerings, populations served, political ideologies, partnerships, regulating agencies, and funding sources. This uniqueness gives organizations autonomy in their operations and pursuits of societal impact. However, this may also lead to “silos” that result from financial and logistical barriers that limit coordinated, integrated service provision across sectors. Furthermore, systems have been designed to oppress and isolate people through policies such as redlining and highway development that disproportionately impact communities of color. This disenfranchisement and fragmentation within systems can breed distrust for government leaders and inefficiencies to reach, engage, serve, support, and treat older adults, which can ultimately disrupt the continuity of care and service delivery and reduce older adults' community participation and social connectedness.

Older adults residing within siloed and fragmented communities are at increased risk of being socially disconnected and not having their social needs met, especially those who experience poorer health, functional or sensory impairments, live alone, or experience additional marginalization (2, 8–11). Because older adults interact with many organizations across sectors for different reasons, these organizations share older adult clients and the responsibility to offer an integrated, coordinated set of “touch points” to address social isolation, loneliness, and general disconnectedness. Misaligned funding streams, competing demands and priorities, and general lack of uniformity across organizations and silos hinder community advancement and the ability to mitigate the health-related consequences of social disconnectedness. However, opportunities exist to bridge silos and narrow societal chasms through purposive collective action that advances research, practice, and policy.

## 3. Opportunities and solutions to strengthen societal and community capacity for social connectedness

A systems approach is needed to reduce societal silos, unify communities, and promote social connectedness among older adults. In this section, we offer nine opportunities and solutions to strengthen and unite communities to improve their cross-sector capacity to meet the social needs of older adults.

### 3.1. Raise awareness about social disconnectedness and advance it as a national priority

The prevalence of social isolation and loneliness among older adults warrants increased recognition as priority public health issues (27, 28). Dedicated awareness-raising efforts are needed to elevate recognition of the risks for, consequences of, and solutions to social disconnectedness among individuals, organizations, and policy makers. Tailored messaging and communication strategies are needed to garner support and buy-in from various stakeholders (30). Although social isolation and loneliness are often discussed and addressed through an individual-level lens, social disconnectedness is also a community-level issue, strongly rooted in social determinants of health framing as well as service and treatment inequities. More efforts are needed to complement and expand the visibility of existing initiatives that are raising awareness about social disconnectedness among older adults and other populations across the life-course [e.g., U.S. Administration for Community Living (ACL)'s Commit to Connect (31), Foundation for Social Connection's Action Forum (32)].

### 3.2. Create a common nomenclature for use across sectors

Similar concepts are phrased and defined differently across disciplines, organizations, and community sectors. As such, it is important to identify commonly used terms and work within communities to establish a consistent terminology surrounding social disconnectedness. Creating a common nomenclature can reduce misunderstandings and facilitate efficiency during collaborations and information exchanges (33). For example, a uniform cross-sector taxonomy may be helpful to define risk factors and criteria, services and programs, and statistical methodologies and approaches.

### 3.3. Develop uniform screening across organizations and sectors

Because social disconnectedness can encompass many constructs [e.g., social isolation, loneliness, social networks, and social supports (2)], organizations commonly use different measures, scales, and screening tools to identify risk among older adults. Measures are commonly selected because of the mission of the organization, the clients they serve, and/or the requirements of their funding sources. However, the use of non-standardized measures (or non-standardized cut-points to indicate risk) can hinder a community's ability to document the prevalence of social disconnectedness or demonstrate collective impact when services and programs are offered through different organizations. It is beneficial to develop and routinely administer uniform and robust measures, which can be aligned with larger national and global initiatives for comparative purposes [e.g., inclusion of uniform social isolation and loneliness me asures collected by the Behavioral Risk Factor Surveillance System (BRFSS) (34) and National Health and Nutrition Examination Survey (NHANES) (35)].

### 3.4. Strengthen cross-sectoral referrals and community navigation

Each organization provides their own set of services and programs that address social disconnectedness. As such, the social needs of older adults may not be entirely addressed by any one organization. To ensure continuity of care for older adults across sectors, organizations should communicate about their respective services and resources (36) and establish seamless inter-agency referral criteria and processes. To enhance these referral systems, organizations should utilize trusted community navigators (e.g., community health workers, promotors, social workers, case managers) who understand specific cultural norms and needs, are familiar with community offerings, and can link older adults to appropriate services and programs. Social prescribing models may help older adults identify and access services and supports (37, 38). Further, technological advances may automate these referral and linkage processes and foster innovative community-clinical-industry partnerships (39, 40).

### 3.5. Establish and expand evidence about effective programs and services

Despite a growing recognition of the importance to address social disconnectedness, there are limited evidence-based programs and services shown to reduce social isolation and loneliness. Many of the interventions that have been tested are focused on individual interventions such as therapy, and less data exist about implementation and evaluation of community-wide or society wide interventions, social infrastructure, or policies. More also needs to be known about how inter-generational initiatives and various living arrangements affect loneliness and social isolation and influence interpersonal bonds and community participation (41). Additional efforts are needed to conduct controlled and pragmatic trials to assess the effectiveness of interventions to address social disconnectedness. It will be critical for such trials to integrate systems thinking approaches and consider the societal context within which trials are conducted to ensure aspects of equity, efficacy, replicability, and scalability can be addressed (42). To complement new interventions that specifically address social disconnectedness, existing interventions developed for other purposes should also be evaluated to determine their indirect benefits on social disconnectedness (43–45).

### 3.6. Improve community places and spaces to promote mobility and connectivity

Older adults with impairments (e.g., physical, sensory, cognitive), limited financial resources, or unreliable transportation may have additional difficulty accessing community resources and each other. As such, it is important to consider the built environment and physical infrastructure within a community to promote community-level mobility and connectivity. Inclusive public spaces are critical for all people to interact with one other, gain trust in community leaders, experience cultural activities, and gain a sense of belonging. Libraries, public parks, community gardens, community centers, and other types of social infrastructure are multifaceted and can improve social connectedness while providing many other benefits to individuals and the community (46). All community-level solutions should be developed with the input and participation of community members to ensure their needs, culture, and interests are included, especially those who are marginalized. Connectivity may be especially difficult in rural communities where resources are more geographically dispersed, which highlights the benefits of delivering services in easily accessible locations that are commonly frequented by older adults (e.g., faith-based organizations, senior centers, healthcare offices, commercial businesses) (47). For example, older residents have better connectivity to shared communal life when their built environment integrates civic, religious, and retail buildings with affordable housing (48).

### 3.7. Adopt unified, systems-level models

**Figure 1:**
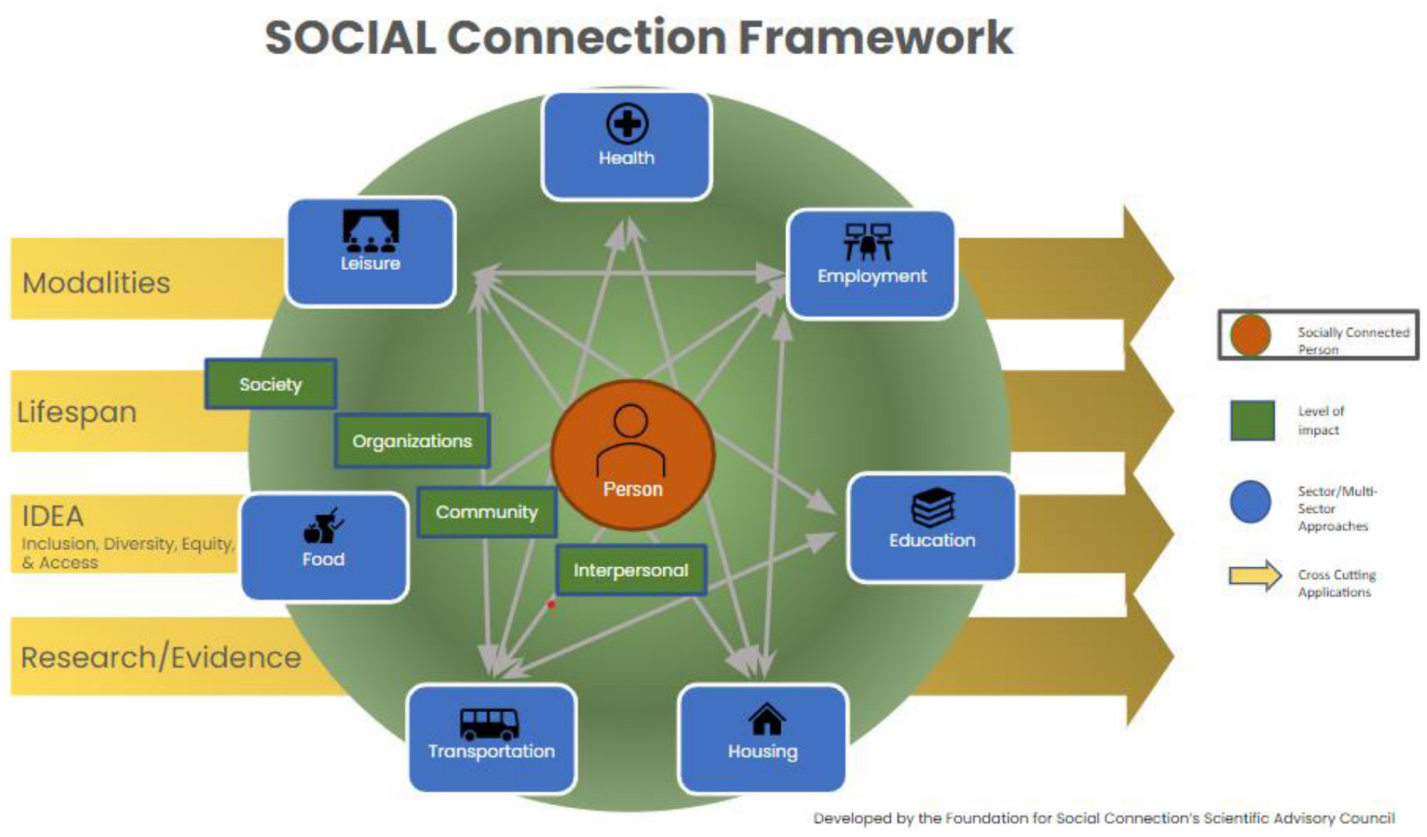
The Systems approach Of Cross-sector Integration and Action across the Lifespan (SOCIAL) Framework (27).

Collective planning across organizations and sectors is often contingent on utilizing a common framework. Such frameworks can help organizations better understand the roles and offerings of other organizations within a community, identify leverage points for collaboration, duplicative services, and service gaps which require additional resources or partnership. An example of an inclusive framework is the Systems approach Of Cross-sector Integration and Action across the Lifespan (SOCIAL) Framework (see Figure 1), which was developed by the Foundation for Social Connection's Scientific Advisory Council (SAC) “to facilitate and accelerate multi-stakeholder actions to reduce social isolation and loneliness, increase social connectedness, and identify opportunities for impact and gaps for additional research and solutions” (27).

### 3.8. Share and leverage funding and data

Funding for research and service provision has become increasingly scarce and competitive in recent years. While organizations rely on their own sources of funding to operate, leveraged funding through strategic partnerships can expand the scope and reach of services beyond the capabilities of any single organization. Public and private funders should consider ways to incentivize community wide collaboration, paying special attention to diversity, equity, and inclusion, to build social connectedness and community participation. Additionally, because each organization collects and generates its own data, efforts are recommended to share and leverage data across organizations and community sectors to alleviate data collection burdens, optimize understanding about older adult clients, and demonstrate collective impact. For example, Health Information Exchanges have been shown to facilitate community partnerships and identify cost savings for programs and services provided to residents (49–52). Another example is the Gravity Project, which defines social determinants of health information so it can be documented in and exchanged across disparate digital health and human service platforms to facilitate payment for social risk data collection and intervention activities (53).

### 3.9. Build inclusive, action-oriented strategic alliances

The formation of community-level coalitions, action alliances, and task forces can unify communities for a common mission. As such, these multi-organization, cross-sector entities can effectively incorporate each of the strategies mentioned above (e.g., raise awareness, create common nomenclature, adopt uniform screening, strengthen referrals, leverage funding). Examples of successful, model entities include the U.S. Coalition to End Social Isolation and Loneliness (CESIL) (54), Building Resilient and Inclusive Communities (BRIC) (55), U.K. Campaign to End Loneliness (56), Australian Ending Loneliness Together (57), and Global Initiative on Loneliness and Connection (GILC) (58).

## 4. Conclusion

Social isolation and loneliness among older adults are growing concerns in many communities across the world. These issues can have a significant impact on an older person's physical and mental health, leading to a decline in overall well-being. To meaningfully combat these problems, communities must recognize collaborative opportunities to address system injustices, transcend sectoral silos, synergize, and leverage efforts for the collective benefit of older adult connectedness.

## Author contributions

All authors listed have made a substantial, direct, and intellectual contribution to the work and approved it for publication.
